# LAT1-Targeting Thermoresponsive Fluorescent Polymer Probes for Cancer Cell Imaging

**DOI:** 10.3390/ijms19061646

**Published:** 2018-06-01

**Authors:** Minami Matsuura, Mariko Ohshima, Yuki Hiruta, Tomohiro Nishimura, Kenichi Nagase, Hideko Kanazawa

**Affiliations:** 1 Faculty of Pharmacy, Keio University, 1-5-30, Shibakoen, Minato-ku, Tokyo 105-8012, Japan; Minami_Matsuura@terumo.co.jp (M.M.); marikoda113@keio.jp (M.O.); nishimura-tm@pha.keio.ac.jp (T.N.); 2 Department of Applied Chemistry, Faculty of Science and Technology, Keio University, 3-14-1 Hiyoshi, Kohoku-ku, Yokohama 223-8522, Japan; hiruta@applc.keio.ac.jp

**Keywords:** poly(*N*-isopropylacrylamide), fluorescent polymer probe, L-type amino acid transporter 1 targeting

## Abstract

L-type amino acid transporter 1 (LAT1) is more highly expressed in cancer cells compared with normal cells. LAT1 targeting probes would therefore be a promising tool for cancer cell imaging. In this study, LAT1-targeting thermoresponsive fluorescent polymer probes based on poly(*N*-isopropylacrylamide-*co*-*N*,*N*-dimethylacrylamide) (P(NIPAAm-*co*-DMAAm)) were synthesized and their affinity for LAT1 was evaluated. The synthesized polymer probes interacted with LAT1 on HeLa cells, and inhibition of l-[^3^H]-leucine, one of the substrates for LAT1 uptake, was investigated. l-Tyrosine-conjugated P(NIPAAm-*co*-DMAAm) inhibited the uptake of l-[^3^H]-leucine, while P(NIPAAm-*co*-DMAAm) and l-phenylalanine-conjugated P(NIPAAm-*co*-DMAAm) did not. This result indicated that l-tyrosine-conjugated polymer has a high affinity for LAT1. The fluorescent polymer probes were prepared by modification of a terminal polymer group with fluorescein-5-maleimide (FL). Above the polymer transition temperature, cellular uptake of the polymer probes was observed because the polymers became hydrophobic, which enhanced the interaction with the cell membrane. Furthermore, quantitative analysis of the fluorescent probe using flow cytometry indicated that l-tyrosine-conjugated P(NIPAAm-*co*-DMAAm)-FL shows higher fluorescence intensity earlier than P(NIPAAm-*co*-DMAAm)-FL. The result suggested that cellular uptake was promoted by the LAT1 affinity site. The developed LAT1-targeting thermoresponsive fluorescent polymer probes are expected to be useful for cancer cell imaging.

## 1. Introduction

The metabolism of cancer cells is different to that of normal cells because of metabolic reprogramming [1]. The alteration of metabolic activity in cancer cells supports the acquisition and maintenance of malignant properties. For example, even when the enzyme is sufficiently supplied, the anaerobic glycolytic system is decidedly increased, and the expression of glucose transporter 1 (GLUT1) is increased to ensure the supply of glucose to the cells [2]. The relationship between altered cellular metabolism and therapeutic outcomes has been reported, and targeting the peculiar metabolism in cancer cells could be an effective strategy for cancer therapy.

Amino acid transporters such as L-type amino acid transporter 1 (LAT1) [3], L-type amino acid transporter 3 (LAT3) [4], system ASC transporter 2 (ASCT2) [5], amino acid transporter system B^0,+^ (ATB^0,+^) [6], and system x_c_^−^ transporter-related protein (xCT) [7] have been reported to increase expression in cancer cells. LAT1 is a plasma membrane transporter that mediates Na^+^-independent transport of neutral amino acids with a bulky lateral chain, such as branched and aromatic amino acids. It appears in normal cells of the placenta and blood-brain barrier to supply the amino acids and is also a transmission route of thyroid hormone and various drugs [1,8]. Many human tumor tissues, such as those of the lung, colon, breast, gliacyte, prostate and pancreas, exhibit enhanced expression of LAT1 [9,10,11,12,13,14]. In addition, such enhanced expression of LAT1 results in poor prognosis, suggesting that malignancy is associated with LAT1 expression.

For substances targeted to transporters whose expression is specifically enhanced in cancer cells, small molecules are used clinically as positron emission tomography (PET) diagnostic agents [15,16,17]. Recently, research into fluorescent probe targeting transporters of cancer cells has been actively conducted and the potential of applying polymers with targeting ability to probes has been established. For example, it has been reported that a multiple ligands-functionalized fluorescent polymer for ASCT2 has a higher affinity than existing ligands [18]. However, as these probes are also recognized by transporters expressed in normal cells, the development of probes with improved selectivity for target cells is necessary. As previously reported, the environments in and around cancer cells can be different compared with those associated with normal cells (e.g., high temperature [1] or low pH [19]) it is, therefore, considered useful to utilize these conditions to trigger responsive behavior in the probe.

Poly(*N*-isopropylacrylamide) (PNIPAAm), which is a temperature responsive polymer [20,21], has been studied in a variety of areas such as high performance liquid chromatography (HPLC) [22,23,24,25,26,27], drug delivery systems (DDS) [28,29,30,31] and materials for regenerative medicine [32,33,34,35,36,37,38]. PNIPAAm changes properties at the lower critical solution temperature (LCST) of 32 °C, being hydrophilic below the LCST and hydrophobic above the LCST. The LCST of the polymer can be tailored to a specific temperature by selective use of the co-monomer used in polymerization. It has been reported that PNIPAAm-FL modified with a fluorescent group at the end of the PNIPAAm chain, is not taken up by cells below the LCST, but does show uptake into cells above the LCST, making it possible to control cellular uptake with temperature [39]. The hydrophilicity/hydrophobicity of the polymer can be varied by controlling not only the temperature, but also the pH [40]. In addition, the fluorescent polymer modified with l-α-phosphatidylethanolamine, dioleoyl (DOPE), which is a membrane fusion lipid, is more rapidly taken up by cells compared with the non-conjugated polymer [41].

**Figure 1:**
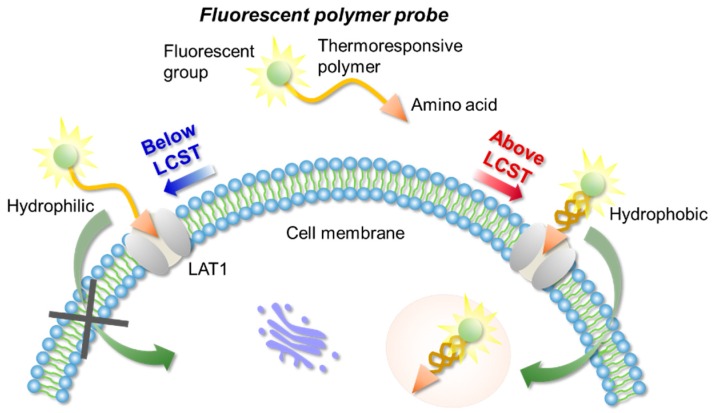
The concept of LAT1-targeting thermoresponsive fluorescent polymer probes.

In this work, we designed a LAT1-targeting fluorescent probe based on a temperature responsive polymer capable of controlling cellular uptake through the temperature of the external environment. The affinity between the polymer probe and LAT1 was evaluated (Figure 1).

## 2. Results and Discussion

### 2.1. Synthesis and Characterization of Polymers

**Scheme 1:**
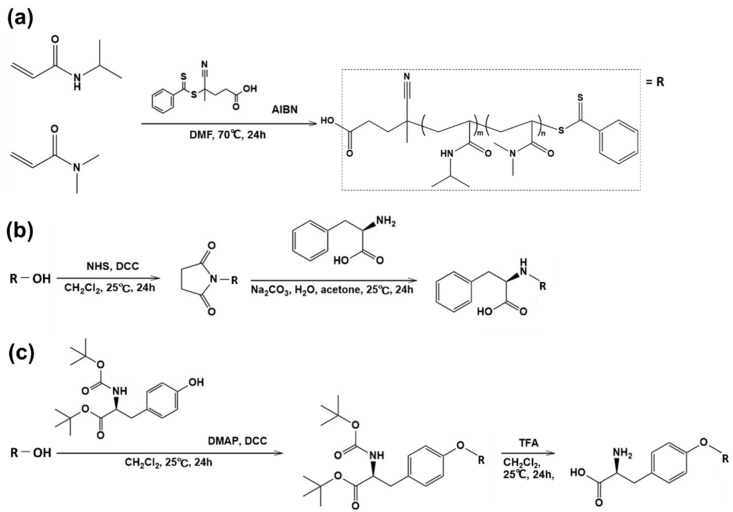
The synthesis of polymers (**a**) P(NIPAAm-*co*-DMAAm); (**b**) Phe-P(NIPAAm-*co*-DMAAm); and (**c**) Tyr-P(NIPAAm-*co*-DMAAm).

**Figure 2:**
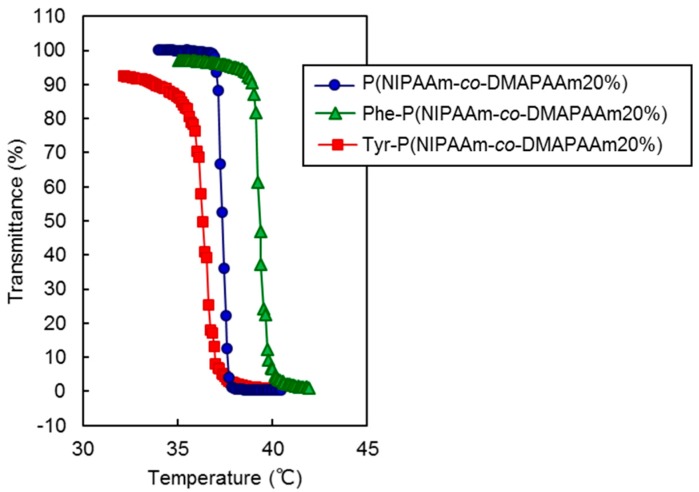
Phase transition profiles of prepared polymers.

The thermoresponsive polymer P(NIPAAm-*co*-DMAAm) was synthesized through reversible addition-fragmentation chain-transfer (RAFT) polymerization (Scheme 1a). The synthesized polymer was characterized by gel permeation chromatography (GPC), observation of the phase transition behavior of the polymer solution (Table 1) and zeta potential (Figure S1). Polyethylene glycol was used as a calibration standard (Figure S2). The averaged molecular weight (M_n_) of the polymers ranged to from 14,200 to 17,700 and decreased as the molar feed composition of DMAAm to NIPAAm was increased. This is because the amount of RAFT agent and radical initiator in the polymerization increased with DMAAm molar composition. The LCST of the prepared polymers increased with increasing DMAAm content because hydrophilicity of the polymer increased with increasing DMAAm composition. l-Phenylalanine-poly(*N*-isopropylacrylamide-*co*-*N*,*N*-dimethylacrylamide) (Phe-P(NIPAAm-*co*-DMAAm)) and l-tyrosine-poly(*N*-isopropylacrylamide-*co*-*N*,*N*-dimethylacrylamide) (Tyr-P(NIPAAm-*co*-DMAAm)) were then obtained by terminal conjugation of the respective amino acids to the polymer (Scheme 1b,c). All amino acid conjugated polymers were found to have LCSTs and exhibited temperature-responsive solubility changes in phosphate buffered saline (PBS), although the LCST was slightly altered by conjugation of the amino acid (Figure 2). These findings indicate that the amino acid conjugated polymer could be applied as a temperature-responsive fluorescent probe, since three polymers with 20 mol % DMAAm have an appropriate LCST near the temperature of the body. As a result, these polymers were used for further investigation.

The polymer zeta potentials were measured in PBS (200 µg/mL) using an ELS-Z2 (Otsuka Electoronics, Osaka, Japan) (Figure S1). The zeta potentials of all polymers were negative and had magnitude; Tyr-P (NIPAAm-*co*-DMAAm20%) < P(NIPAAm-*co*-DMAAm20%) < Phe-P(NIPAAm-*co*-DMAAm20%). This can be attributed to the nature of the functional end groups. P(NIPAAm-*co*-DMAAm20%) and Phe-P(NIPAAm-*co*-DMAAm20%) have a carboxyl group in the terminal polymer end group, while Tyr-P(NIPAAm-*co*-DMAAm20%) has a carboxyl group and an amino group. These functional group differences lead to different zeta potentials.

### 2.2. Inhibition of l-[^3^H]-Leucine Uptake

**Figure 3:**
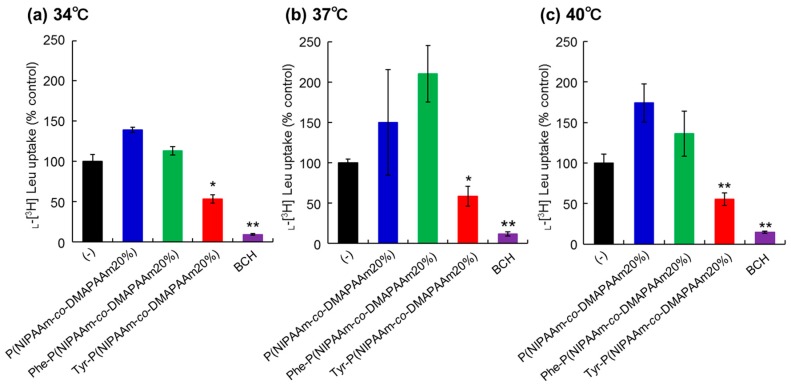
Inhibition of l-[^3^H]-leucine uptake in HeLa cells at (**a**) 34 °C; (**b**) 37 °C and (**c**) 40 °C. The concentration of inhibitors P(NIPAAm-*co*-DMAAm20%, Phe-P(NIPAAm-*co*-DMAAm20%), and Tyr-P(NIPAAm-*co*-DMAAm20%), and system L specific inhibitor, BCH was 2 mM. * *p* < 0.05, ** *p* < 0.01 vs. control (*n* = 3).

To investigate the interaction between the prepared polymers and LAT1 on cancer cells, uptake of l-[^3^H]-leucine, one of the substrates for LAT1, was observed in the presence of the polymers or 2-aminobicyclo-(2,2,1)-heptane-2-carboxylic acid (BCH), a LAT1 inhibitor (Figure 3). HeLa cells were used as model cancer cells as LAT1 expression on HeLa cells was confirmed by western blotting and immunostaining (Figure S3) [42]. In the present study, we did not confirm the expression of other types of L-type amino acid transporters in Hela cells such as LAT2 or LAT3. However, expression of LAT2 and LAT3 is relatively low compared to that of LAT1 [43,44]. Thus, we consider that the LAT1 dominantly uptake of leucine compared to other types of transporters. To verify the difference in the inhibitory effect due to changes in the physical properties of the polymers with temperature change, measurements were made at 34 °C, 37 °C, and 40 °C. HeLa cells incubated with P(NIPAAm-*co*-DMAAm20%) exhibited l-[^3^H]-leucine uptake at all temperatures. This result indicated that P(NIPAAm-*co*-DMAAm20%) without conjugated amino acids did not inhibit leucine uptake through LAT1. In addition, P(NIPAAm-*co*-DMAAm20%) with l-phenylalanine did not inhibit the uptake of l-[^3^H]-leucine. In contrast, P(NIPAAm-*co*-DMAAm20%) with l-tyrosine inhibited the uptake of l-[^3^H]-leucine. In the polymer modified with l-phenylalanine as the end group, only the α-carboxyl group exists, whereas the polymer in which l-tyrosine is the end group has an α-amino group and α-carboxyl group. Given that it has been reported that the positive charge of the α-amino group and the negative charge of the α-carboxyl group are involved in substrate recognition of LAT1 [45], the results appear to be consistent with previous findings. The inhibitory effect of the Tyr-P(NIPAAm-*co*-DMAAm20%) did not differ greatly as a result of temperature variation. Therefore, regardless of the change in the physical properties (hydrophilic/hydrophobic) of the polymer, LAT1 can be considered to recognize the terminal l-tyrosine.

### 2.3. Cellular Uptake of Fluorescent Probes

**Scheme 2:**
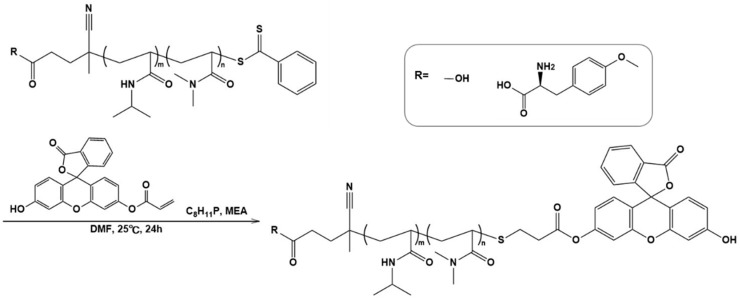
Synthesis of fluorescent polymer probes.

**Figure 4:**
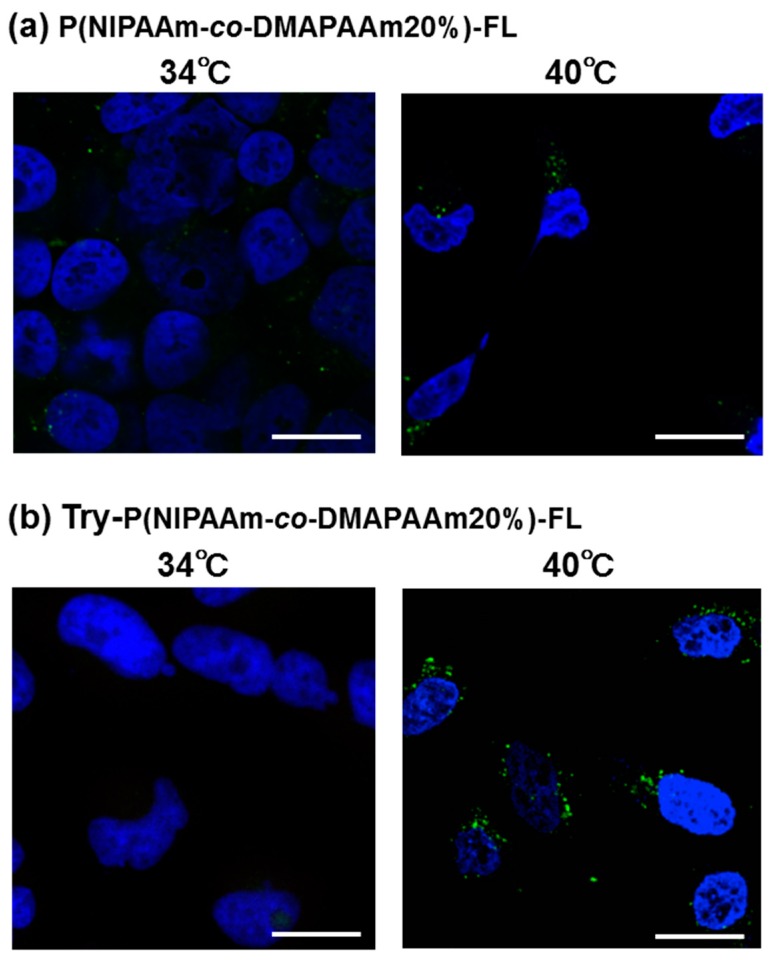
Microscopy images of HeLa cells after 4 h treatment with (**a**) P(NIPAAm-*co*-DMAAm20%)-FL and (**b**) Tyr-P(NIPAAm-*co*-DMAAm20%)-FL at 34 °C and 40 °C. The fluorescent probes appear green and the nuclei were stained blue (DAPI). Magnification is 40×; the scale bar represents 20 μm.

**Figure 5:**
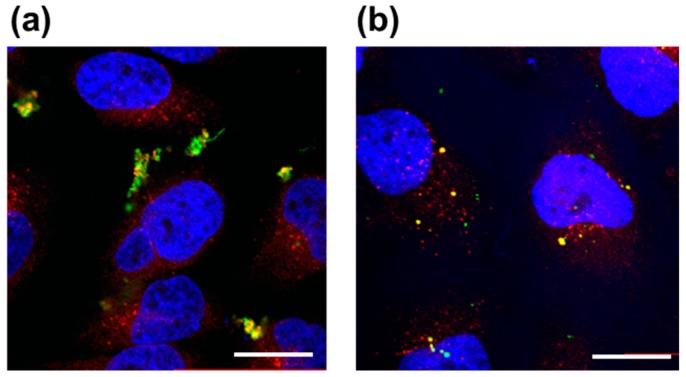
Confocal laser scanning microscopy images of HeLa cells after 4 h treatment with (**a**) P(NIPAAm-*co*-DMAAm20%)-FL and (**b**) Tyr-P(NIPAAm-*co*-DMAAm20%)-FL at 40 °C. Fluorescent probes appear green, lysosomes are stained red (LysoTracker Red DND-99), and the nuclei are stained blue (DAPI). Magnification is 40×; the scale bar represents 20 μm.

Fluorescent polymer probes were prepared by conjugating fluorescein-5-maleimide to the end group of the polymers (Scheme 2). The characterization of the prepared fluorescent polymer probes is described in the Supplementary Materials. First, the influence of incubation temperature and LAT1 recognition on the cellular uptake of the fluorescent probes was investigated. P(NIPAAm-*co*-DMAAm)-FL or Tyr-P(NIPAAm-*co*-DMAAm)-FL was added to HeLa cells, which were then incubated for 4 h below the LCST; 34 °C or above the LCST; 40 °C. After incubation, fluorescence microscopy, and flow cytometry were performed. Fluorescence microscopy showed that below the LCST, the fluorescent probe adsorbed to the cell membrane and cellular uptake was not confirmed (Figure 4). In contrast, above the LCST, cellular uptake of fluorescent probes was confirmed. This is attributed to the hydrophilic/hydrophobic change of the fluorescent polymer probe. Below the LCST the polymer probe is hydrophilic and its affinity with the cell membrane is low. Thus, the fluorescent probe was not taken up by the cells. However, when the polymer became hydrophobic above the LCST, the affinity with the cell membrane was enhanced and cellular uptake increased. Unlike P(NIPAAm-*co*-DMAAm20%)-FL, it was confirmed that Tyr-P(NIPAAm-*co*-DMAAm20%)-FL uniformly incorporated into cells above the LCST (Figure 4). To confirm the localization of the fluorescent probes within the cells, lysosome staining was carried out using LysoTracker Red DND-99 (Figure 5). The confocal microscopy image indicates that the fluorescent probes co-localized with the lysosome. These results demonstrate that endocytosis of the fluorescent probes occurred, which is attributed to the fluorescent polymer probes becoming hydrophobic and interacting with the cell membrane.

**Figure 6:**
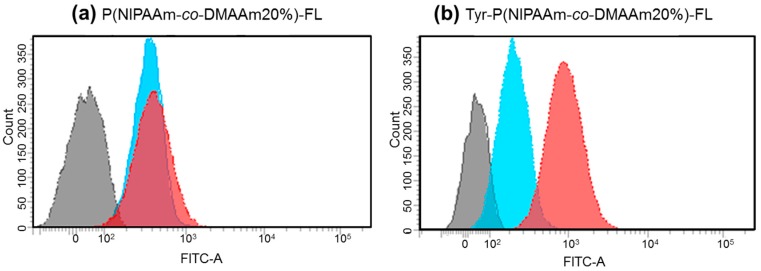
Representative flow cytometry histograms of fluorescence intensity of HeLa cells after 4 h incubation with (**a**) P(NIPAAm-*co*-DMAAm20%)-FL and (**b**) Tyr-P(NIPAAm-*co*-DMAAm20%)-FL at 34 °C (blue) and 40 °C (red).

**Figure 7:**
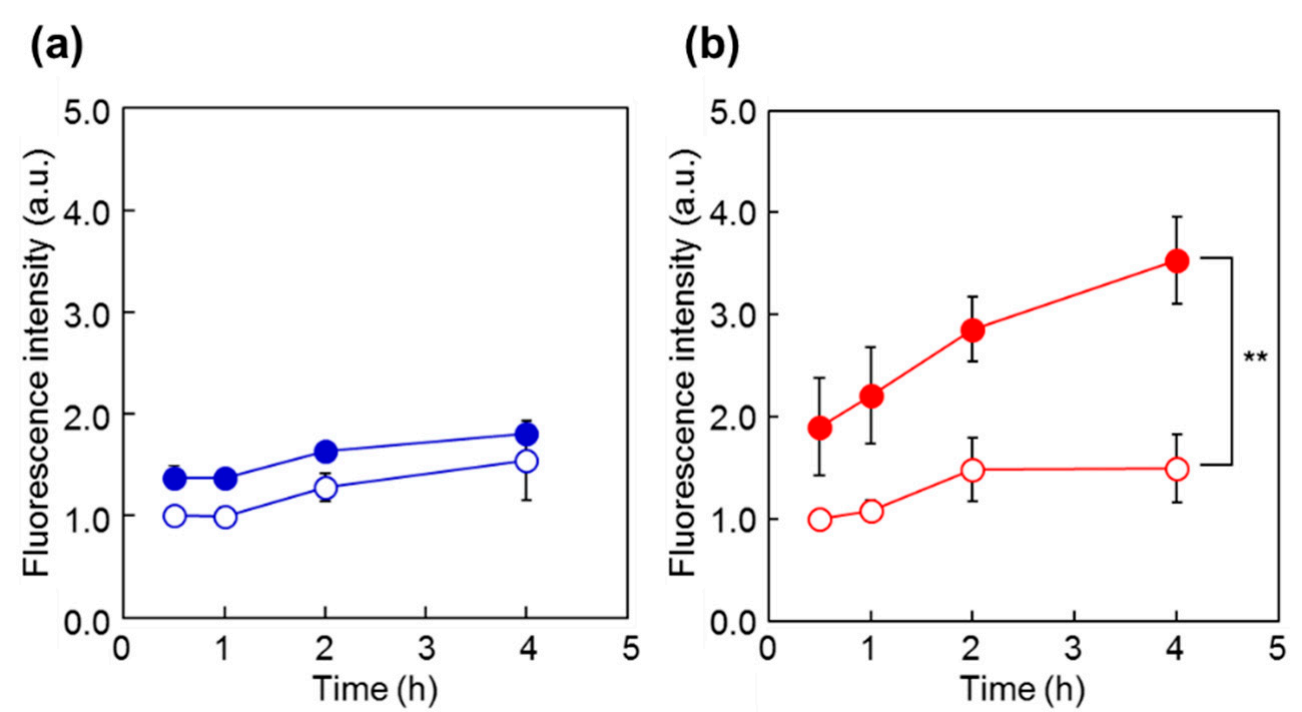
Fluorescence intensity of HeLa cells at various time points following incubation with polymer probes. Cells were incubated with the probes for 0.5, 1, 2, or 4 h ((**a**) P(NIPAAm-*co*-DMAAm20%)-FL; (**b**) Tyr-P(NIPAAm-*co*-DMAAm20%)-FL) at either 40 °C (close circles) or 34 °C (open circles). Data are the mean ± S.D. (*n* = 3) ** *p* < 0.01.

To investigate the cellular uptake behavior of the polymer probes, the fluorescence intensity of HeLa cells was analyzed by flow cytometry after incubation with the probes at 34 °C and 40 °C (Figure 6). For both P(NIPAAm-*co*-DMAAm20%)-FL and Tyr-P(NIPAAm-*co*-DMAAm20%)-FL, constant fluorescence intensities were detected below the LCST. However, this is probably owing to the adsorption of the fluorescent probe onto the cell membrane. After 4 h, cells incubated with Tyr-P(NIPAAm-*co*-DMAAm20%)-FL showed a marked increase in fluorescence intensity above the LCST compared with that of cells incubated with P(NIPAAm-*co*-DMAAm20%)-FL, owing to the affinity between the terminal l-tyrosine of the polymer probe and LAT1 on the HeLa cells. In addition, to investigating the time-dependent cellular uptake of the polymer probe, the fluorescence intensity of the HeLa cells was measured over time (Figure 7). In the case of P(NIPAAm-*co*-DMAAm20%)-FL, no significant difference in fluorescence intensity was observed between cells incubated above and below the LCST; although the fluorescence intensity of cells incubated above the LCST was slightly higher than that of those incubated below the LCST (Figure 7a). This result indicates that the hydrophobicity change of the fluorescent polymer probe was insufficient to induce uptake of the probe into cells. In contrast, in the case of Tyr-P(NIPAAm-*co*-DMAAm20%)-FL, two times greater fluorescence intensity was observed for cells incubated above the LCST compared with those incubated below the LCST, even after 30 min of incubation (Figure 7b). Furthermore, the fluorescence intensity of cells incubated above the LCST was three times greater compared with that of cells incubated below the LCST after 4 h of incubation. The results also suggested that the synergetic effect of the hydrophobicity of the polymer probe and the affinity between LAT1 and terminal l-tyrosine, promote cellular uptake of the polymer probe. 

**Figure 8:**
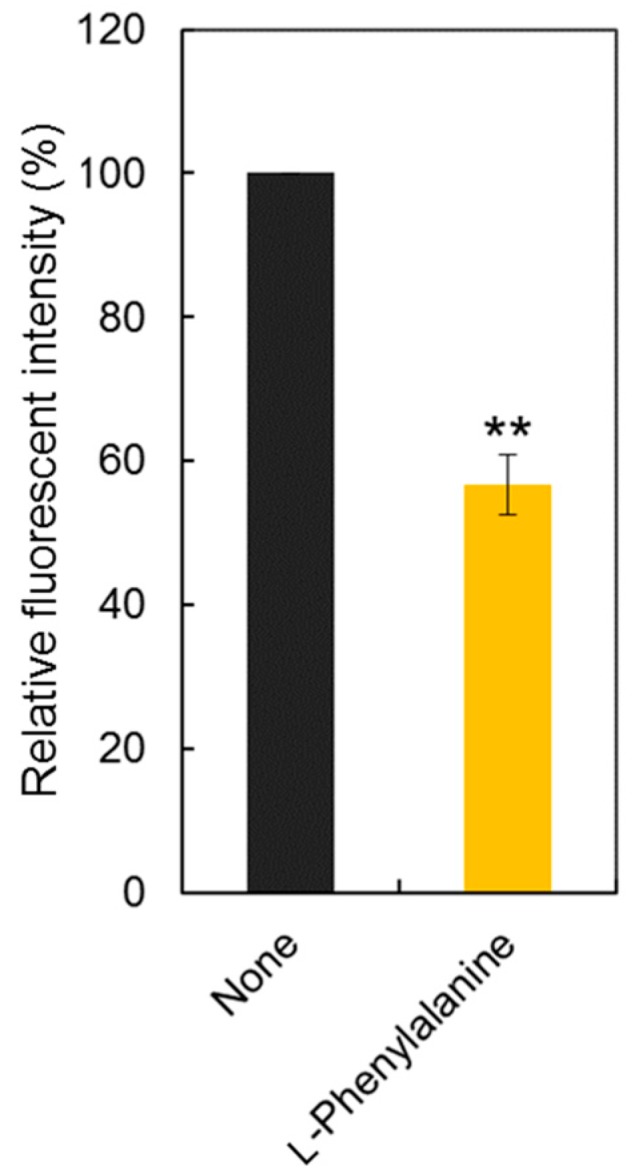
Comparison of fluorescence intensity with/without l-phenylalanine (30 mM). Data are the mean ± S.D. (*n* = 3) ** *p* < 0.01.

To confirm the LAT1 engagement on cellular uptake of Tyr-P(NIPAAm-*co*-DMAAm20%)-FL, uptake of the polymer probe was observed following incubation in the presence of l-phenylalanine (Figure 8). l-Phenylalanine was used as the competing agent rather than l-tyrosine because the hydroxyl group of l-tyrosine was used for conjugation to the polymer end group, making the chemical structure of conjugated l-tyrosine more similar to l-phenylalanine than to l-tyrosine. In this investigation, the concentration of l-phenylalanine was set at 5 mg/mL based on a previous report [46]. The fluorescence intensity was found to be lower than that measured for cells not treated with l-phenylalanine, indicating that LAT1 mediator was involved in the cellular uptake of Tyr-P(NIPAAm-*co*-DMAAm20%)-FL.

The developed fluorescent polymer probe exhibited affinity with LAT1 and temperature-dependent cellular uptake. The fluorescent polymer probe would therefore be useful for cancer cell imaging that responds to the malignancy associated with LAT1 expression and the environment of cancer cells.

## 3. Materials and Methods

### 3.1. Materials

*N*-Isopropylacrylamide (NIPAAm) was kindly provided by KJ Chemicals (Tokyo, Japan) and purified by recrystallization from *n*-hexane. *N*,*N*-Dimethylacrylamide (DMAAm) and 2,2′-Azobisisobutyronitrile (AIBN) were obtained from Wako Pure Chemical Industry (Osaka, Japan) and purified by distillation and recrystallization, respectively. 4-Cyano-4-(phenylcarbonothioylthio)pentanoic acid and fluorescein-5-maleimide were obtained from Sigma-Aldrich (St. Louis, MO, USA). l-Phenylalanine and l-tyrosine were purchased from Peptide Institute (Tokyo, Japan). *N*,*N*′-Dicyclohexylcarbodiimide (DCC) was purchased from Kanto Chemicals (Tokyo, Japan). *N*-Hydroxysuccinimide (NHS) was obtained from Merck Japan (Tokyo, Japan). Water was purified using a PURELAB FLEX (Organo, Tokyo, Japan).

### 3.2. Synthesis of Polymers

#### 3.2.1. P(NIPAAm-*co*-DMAAm)

Thermoresponsive polymers with LCST near body temperature were synthesized via RAFT polymerization of NIPAAm and DMAAm. A typical polymerization procedure of P(NIPAAm-*co*-DMAAm) with 12.5% DMAAm feed composition was as follows; NIPAAm (5.0 g, 44 mmol) and DMAAm (0.6 g, 6.1 mmol) were dissolved in DMF (15 mL). AIBN (31 mg, 19 mmol) and 4-cyano-4-(phenylcarbonothioylthio)pentanoic acid (105 mg, 0.6 mmol) were added to the solution as the radical initiator and RAFT agent, respectively. The reaction mixture was degassed by bubbling with nitrogen gas for 20 min and the polymerization was allowed to proceed at 70 °C for 24 h. After the polymerization, the solution was added dropwise to diethyl ether to precipitate the synthesized polymer. The precipitate was further purified by repeated precipitation using diethyl ether from a solution of acetone, filtered, and dried in vacuo to give P(NIPAAm-*co*-DMAAm12.5%) as a white solid (2.5 g). Using the same procedure, P(NIPAAm-*co*-DMAAm20%) (2.9 g) was obtained from NIPAAm (5.0 g, 44 mmol), DMAAm (1.1 g, 11 mmol), AIBN (33 mg, 20 mmol) and 4-cyano-4-(phenylcarbonothioylthio)pentanoic acid (114 mg, 0.7 mmol). P(NIPAAm-*co*-DMAAm35%) (3.0 g) was obtained from NIPAAm (5.0 g, 44 mmol), DMAAm (2.4 g, 24 mmol), AIBN (40 mg, 25 mmol), and 4-cyano-4-(phenylcarbonothioylthio)pentanoic acid (137 mg, 0.8 mmol).

#### 3.2.2. Phe-P(NIPAAm-*co*-DMAAm)

P(NIPAAm-*co*-DMAAm12.5%) (1.0 g, 0.07 mmol) was dissolved in dichloromethane (7 mL). NHS (19.2 mg, 0.17 mmol) and DCC (34.4 mg, 0.17 mmol) were added to the polymer solution, and the reaction was allowed to proceed at room temperature for 24 h. After removal of the precipitated dicyclohexylurea by filtration, the crude product was further purified using diethyl ether, filtered, and dried. NHS-P(NIPAAm-*co*-DMAAm12.5%) was obtained as a white solid. l-Phenylalanine (27.5 mg, 0.17 mmol) and Na_2_CO_3_ (17.7 mg, 0.17 mmol) were dissolved in H_2_O (3 mL), and the NHS-P(NIPAAm-*co*-DMAAm12.5%) was dissolved in the solution. Acetone (7.5 mL) was then added to the solution and the reaction was allowed to proceed with stirring at 25 °C for 24 h. After the reaction, the acetone was evaporated, the residual product was removed by filtration, and the filtrate was purified by dialysis using a dialysis membrane with a 3500 molecular-weight cut-off (Spectra/Por, Spectrum Laboratories, Rancho Dominguez, CA, USA). After purification of the polymer solution, Phe-P(NIPAAm-*co*-DMAAm12.5%) (600 mg) was obtained as a white solid. Using the same procedure, Phe-P(NIPAAm-*co*-DMAAm20%) (545 mg) was obtained from P(NIPAAm-*co*-DMAAm20%) and Phe-P(NIPAAm-*co*-DMAAm35%) (588 mg) was obtained from P(NIPAAm-*co*-DMAAm35%).

#### 3.2.3. Tyr-P(NIPAAm-*co*-DMAAm)

l-Tyrosine was conjugated to P(NIPAAm-*co*-DMAAm12.5%). The protection of the amino and carboxyl group of l-tyrosine, *tert*-butyl (*tert*-butoxycarbonyl)-l-tyrosine, was performed according to a previously reported method [47]. P(NIPAAm-*co*-DMAAm12.5%) (1.0 g, 0.07 mmol) was dissolved in dichloromethane (4 mL). *tert*-Butyl (*tert*-butoxycarbonyl)-l-tyrosine (40 mg, 0.13 mmol), DMAP (16 mg, 0.13 mmol), and DCC (69 mg, 0.33 mmol) were added to the polymer solution and the reaction was allowed to proceed at room temperature for 24 h. After the reaction, the polymer solution was precipitated in diethyl ether, and *tert*-butyl (*tert*-butoxycarbonyl)-l-tyrosine-P(NIPAAm-*co*-DMAAm12.5%) (880 mg) was obtained as a yellow solid. Deprotection of *tert*-butyl and *tert*-butoxycarbonyl was then performed. *tert*-Butyl (*tert*-butoxycarbonyl)-l-tyrosine-P(NIPAAm-*co*-DMAAm12.5%) was dissolved in dichloromethane (3 mL) and trifluoroacetic acid (2 mL) was added to the solution. Deprotection was performed at room temperature for 24 h. After the reaction, the solution was dialyzed using a dialysis membrane with a 3500 molecular-weight cut-off. The crude product was further purified by repeated precipitation using hexane (300 mL), filtered, and dried to provide Tyr-P(NIPAAm-*co*-DMAAm12.5%) (477 mg). Using the same procedure, Tyr-P(NIPAAm-*co*-DMAAm20%) (491 mg) was obtained from P(NIPAAm-*co*-DMAAm20%), and Tyr-P(NIPAAm-*co*-DMAAm35%) (462 mg) was obtained from P(NIPAAm-*co*-DMAAm35%).

### 3.3. Characterization of Polymers

#### 3.3.1. Gel Permeation Chromatography

Molecular weight was measured using a GPC system (GPC-8020, Tosoh, Tokyo, Japan). A TSK guard column, and two TSK GEL α-M columns were used. The mobile phase was DMF containing 10 mM LiCl with a flow rate of 1.0 mL/min. Samples were dissolved in mobile phase (0.5 mg/mL). The column temperature was 40 °C. Calibration was performed using polyethylene glycol standards (Tosoh) (Figure S1).

#### 3.3.2. Phase Transition Behavior of Polymers

The LCST of the polymers was determined by measuring the optical transmittance in PBS solution (0.5 *w*/*v* %) over a temperature range using a UV–VIS spectrophotometer (V-630, Jasco, Tokyo, Japan). The transmittance of the solution was measured at 500 nm. The temperature was controlled using a PT-31 Peltier system (Krüss, Hamburg, Germany) and an ETC-717 controller (Jasco). The heating rate was 0.1 °C/min. The LCST was determined to be the temperature of 50% transmittance of the solution.

### 3.4. Cell Culture

HeLa and HEK 293 cells (RIKEN BRC Cell Bank, Tsukuba, Japan) were cultured in MEM (Thermo Fisher, Waltham, MA, USA), supplemented with 10% fetal bovine serum (FBS; Bioserum, Victoria, Australia), 50 units/mL penicillin, 50 μg/mL streptomycin, and 146 μg/mL l-glutamine, at 37 °C under 5% CO_2_. Cells were cultured for 2–5 days to achieve approximately confluent conditions before performing all experiments.

### 3.5. Inhibition of l-[^3^H] Leucine Uptake

HeLa cells were seeded in 24-well plates at a density of 1.0 × 10^5^ cells per well, in 1 mL of medium. After overnight incubation, the plates were placed on the dry bath incubator (Nippon Genetics Europe, Dueren, Germany) heating at 34 °C, 37 °C, or 40 °C. After removal of the medium, the cells were rinsed with warmed buffer (125 mM NaCl, 4.8 mM KCl, 5.6 mM d-(+)-Glucose, 1.2 mM CaCl_2_·H_2_O, 1.2 mM KH_2_PO_4_, 1.2 mM MgSO_4_ and 25 mM HEPES), and pre-incubated with warmed buffer for 10 min. The cells were incubated with 1 µCi/mL l-[^3^H]-leucine (Moravek Biochemicals, Richland, WA, USA) and polymer or BCH (Sigma-Aldrich) (2 mM) as the inhibitor, for 10 min. The cells were rinsed with cooled buffer and added to 0.1 M NaOH (250 μL) at 4 °C overnight. Following the overnight incubation, 0.1 M HCl (250 μL) was added to each well, and the sample solutions (400 μL) were dissolved in liquid scintillation solvent (Clear-sol I, Nacalai Tesque, Kyoto, Japan) in a vial. l-[^3^H]-Leucine was detected using a Packard Tri-Carb 3170 TR/SL liquid scintillation analyzer (PerkinElmer Japan, Kanagawa, Japan). The protein concentrations in the samples were determined using a Pierce BCA Protein Assay Reagent Kit.

### 3.6. Synthesis of Fluorescent Probes

P(NIPAAm-*co*-DMAAm20%) (50 mg, 3.3 µmol) was dissolved in tetrahydrofuran (THF; 2 mL). 2-Aminoethanol (2.0 μL, 3.3 × 10^−2^ mmol) dissolved in THF (1 mL) was then added dropwise and purged with nitrogen for 30 min. After confirming visually that the solution became colorless from yellow, fluorescein-5-maleimide (2.9 mg, 6.7 μmol) dissolved in THF (1 mL) was added and the reaction was allowed to proceed under a nitrogen atmosphere for 24 h. The resulting residue was purified by dialysis using a dialysis membrane with a 3500 molecular-weight cut-off at 4 °C for three days. The solvent was evaporated to condense the polymer solution, then the solution was added dropwise to diethyl ether to precipitate the polymer. The precipitate was filtered, and dried in vacuo. P(NIPAAm-*co*-DMAAm20%)-FL (41.8 mg) was obtained as pale-yellow solid. Tyr-P(NIPAAm-*co*-DMAAm20%)-FL (57.2 mg) was synthesized from Tyr-P(NIPAAm-*co*-DMAAm20%) according to the same procedure. The modification rate of the fluorescein-5-maleimide to polymer was determined by absorbance of the fluorescent group.

### 3.7. Cellular Uptake of Fluorescent Probes

#### 3.7.1. Confocal Microscopy of Cellular Uptake

HeLa cells were seeded in a BioCoat™ Collagen I Cellware four-well culture slide (Corning, Corning, NY, USA) at a density of 1.0 × 10^5^ cells per well, in 0.5 mL of medium. After overnight incubation, the cells were further incubated for 4 h with fluorescent probes (1.6 × 10^−2^ mM) at 34 °C or 40 °C in a humidified atmosphere containing 5% CO_2_. After incubation, the cells were rinsed twice with 1 mM ethylenediaminetetraacetic acid (EDTA) in PBS, and 50 μM LysoTracker Red in MEM was added. Cells were then incubated for 30 min at 37 °C. The cells were rinsed twice with 1 mM EDTA in PBS and fixed with 4% paraformaldehyde in PBS for 20 min. After cells were rinsed twice with PBS, coverslips were mounted over cells in VECTORSHIELD^®^ Hard Set™ Mounting Medium with DAPI (Vector Labolatories, Burlingame, CA, USA). The cells were then observed with a FV1000D confocal laser-scanning microscope (Olympus, Tokyo, Japan).

#### 3.7.2. Flow Cytometry Analysis of Cellular Uptake

HeLa cells were seeded in six-well plates at a density of 2.0 × 10^5^ cells per well, in 2 mL of cell medium. After overnight incubation, the cells were further incubated for 0.5, 1, 2, or 4 h with fluorescent probes (1.6 × 10^−2^ mM) at 34 °C or 40 °C in a humidified atmosphere containing 5% CO_2_. Following incubation the medium was aspirated, and cells were rinsed twice with 1 mM EDTA in PBS. The cells were then trypsinized, and rinsed with MEM supplemented with 10% FBS. The cells were rinsed twice with 1 mM EDTA in PBS, followed by PBS, filtered through 35 μm nylon mesh, and finally examined on a LSR II flow cytometer (Becton-Dickinson Biosciences, San Jose, CA, USA). Fluorescence histograms and dot plots were generated using Cell Quest software (for figures, histograms were recreated using FACSDiVa software (Becton-Dickinson Biosciences)).

#### 3.7.3. Cell Uptake Inhibition with Amino Acid

HeLa cells were seeded in 6-well plates at a density of 2.0 × 10^5^ cells per well, in 2 mL of cell medium. After overnight incubation, the cells were incubated for a further 4 h with fluorescent probe (1.6 × 10^−^^2^ mM) and l-phenylalanine (30 mM), which are substrates of LAT1, at 40 °C in a humidified atmosphere containing 5% CO_2_. Following incubation, the procedure was the same as in Section 3.7.1.

### 3.8. Statistical Analysis

Student’s non-paired *t*-test was used to compare the normally distributed values between the groups. *p* < 0.05 was considered statistically significant.

## 4. Conclusions

In this study we succeeded in developing a fluorescent polymer probe, which is recognized by LAT1 and is taken up into cells in response to temperature, with high affinity for cancer cells. Intracellular uptake inhibition experiments with l-[^3^H]-leucine in HeLa cells showed that Tyr-P(NIPAAm-*co*-DMAAm) inhibited uptake of l-[^3^H]-leucine. It was suggested that Tyr-P(NIPAAm-*co*-DMAAm) was recognized by LAT1 owing to the presence of both of the terminal amino and carboxyl groups. The fluorescent polymer probe, Tyr-P (NIPAAm-*co*-DMAAm)-FL, was prepared by conjugating fluorescein-5-maleimide to the end group of the polymer, and was used for fluorescence microscopy and flow cytometry experiments with HeLa cells. Below the LCST the fluorescent probe was adsorbed by the cell membrane and cellular uptake was not confirmed. In contrast, above the LCST, cellular uptake of the fluorescent probe was confirmed. Flow cytometry analysis showed that above the LCST the fluorescence intensity of cells incubated with Tyr-P(NIPAAm-*co*-DMAAm20%)-FL was greater than that of cells incubated below the LCST. These results suggest that endocytosis of the fluorescent polymer probe occurred when the probes became hydrophobic and interacted with the cell membrane.

The LAT1-targeting thermoresponsive fluorescent polymer probes are expected to show high cancer selectivity owing to high LAT1 affinity and can regulate intracellular uptake by changes in hydrophilicity/hydrophobicity depending on temperature.

## Figures and Tables

**Table 1:** Polymer characterization.

	Terminal Group	M_n_^a^	M_w_^a^	M_w_/M_n_^a^	LCST (°C) ^b^
P(NIPAAm-*co*-DMAAm12.5%)	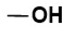	17,700	22,100	1.25	35.1
P(NIPAAm-*co*-DMAAm20%)		16,700	19,500	1.17	37.3
P(NIPAAm-*co*-DMAAm35%)		14,200	16,400	1.15	47.1
Phe-P(NIPAAm-*co*-DMAAm12.5%)	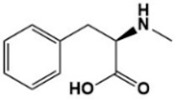	18,500	21,800	1.18	35.7
Phe-P(NIPAAm-*co*-DMAAm20%)		18,200	22,400	1.23	39.3
Phe-P(NIPAAm-*co*-DMAAm35%)		16,600	19,500	1.17	49.4
Tyr-P(NIPAAm-*co*-DMAAm12.5%)	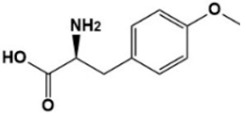	13,400	16,100	1.20	34.1
Tyr-P(NIPAAm-*co*-DMAAm20%)		12,100	15,600	1.29	36.3
Tyr-P(NIPAAm-*co*-DMAAm35%)		12,700	14,500	1.14	46.2

^a^ Determined by GPC. ^b^ Determined by temperature dependent optical transmittance change.

## Supplementary Materials

Supplementary materials can be found at http://www.mdpi.com/1422-0067/19/6/1646/s1.

## Abbreviations

AIBN	2,2′-Azobisisobutyronitrile
ASCT2	System ASC transporter 2
ATB^0,+^	Amino acid transporter system B^0,+^
BCH	2-Aminobicyclo-(2,2,1)-heptane-2-carboxylic acid
DCC	*N*,*N*′-Dicyclohexylcarbodiimide
DDS	Drug delivery system
DMAAm	*N*,*N*-Dimethylacrylamide
DOPE	l-α-Phosphatidylethanolamine, dioleoyl
EDTA	Ethylenediaminetetraacetic acid
FBS	Fetal bovine serum
FL	Fluorescein-5-maleimide
GLUT1	Glucose transporter 1
HPLC	High performance liquid chromatography
LAT1	L-type amino acid transporter 1
LAT3	L-type amino acid transporter 3
LCST	Lower critical solution temperature
NHS	*N*-Hydroxysuccinimide
NIPAAm	*N*-Isopropylacrylamide
PBS	Phosphate buffered saline
PET	Positron emission tomography
Phe-P(NIPAAm-*co*-DMAAm)	l-Phenylalanine-poly(*N*-isopropylacrylamide-*co*-*N*,*N*-dimethylacrylamide)
PNIPAAm	Poly(*N*-isopropylacrylamide)
P(NIPAAm-*co*-DMAAm)	Poly(*N*-isopropylacrylamide-*co*-*N*,*N*-dimethylacrylamide)
THF	Tetrahydrofuran
Tyr-P(NIPAAm-*co*-DMAAm)	l-Tyrosine-poly(*N*-isopropylacrylamide-*co*-*N*,*N*-dimethylacrylamide)
xCT	System xc-transporter-related protein
