# Supplementary material for: LAT1-Targeting Thermoresponsive Fluorescent Polymer Probes for Cancer Cell Imaging
Source: Int J Mol Sci. 2018 Jun 1;19(6):1646. doi: 10.3390/ijms19061646 (PMC6032285; doi:10.3390/ijms19061646)
Supplement: Supplementary file 1 [file ijms-19-01646-s001.pdf]

Supplementary Materials

LAT1-Targeting Thermoresponsive Fluorescent Polymer Probes for Cancer Cell Imaging

Minami Matsuura [1], Mariko Ohshima [1], Yuki Hiruta [2], Tomohiro Nishimura [1], Kenichi Nagase [1,*] and Hideko Kanazawa [1,*]

[1] Faculty of Pharmacy, Keio University, 1-5-30, Shibakoen, Minato-ku, Tokyo 105-8012, Japan; Minami_Matsuura@terumo.co.jp (M.M.); marikoda113@keio.jp (M.O.); nishimura-tm@pha.keio.ac.jp (T.N.)

[2] Department of Applied Chemistry, Faculty of Science and Technology, Keio University, 3-14-1 Hiyoshi, Kohoku-ku, Yokohama 223-8522, Japan; hiruta@applc.keio.ac.jp (Y.H.)

* Correspondence: nagase-kn@pha.keio.ac.jp (K.N.); kanazawa-hd@pha.keio.ac.jp (H.K.); Tel.: +81-3-5400-1378 (K.N.); Tel.: +81-3-5400-2684 (H.K.)

Zeta Potential Measurement

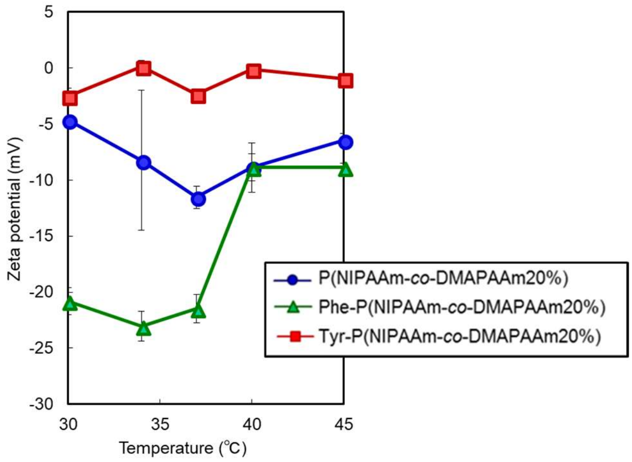

Figure S1. Zeta potential profiles of prepared polymers.

Calibration of Gel Permeation Chromatography

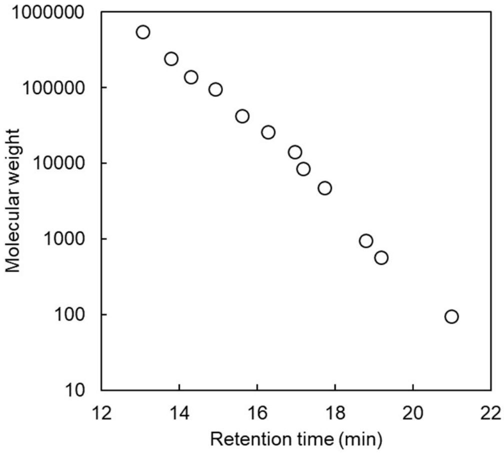

Figure S2. GPC calibration curve using polyethylene glycol standard.

Western Blotting and Immunofluorescence

HeLa and HEK 293 cells were homogenized in lysis buffer (1 M Tris-HCl (pH 7.4), 5 N NaCl, 500 nM EDTA, 20% TritonX-100 and complete mini-EDTA-free) with 5 strokes for 10 min on ice. Lysates were centrifuged at 16,000×*g* at 4 °C for 10 min, and protein concentrations were determined using a Pierce BCA Protein Assay Reagent Kit (Thermo Scientific, Rockford, IL). The total lysates were resuspended in lysis buffer and treated with 5×SDS-sample buffer (10% sodium dodecyl sulfate, 25% 2-mercaptoethanol, 250 mM Tris-HCl (pH 7.4) and 50% glycerol). The protein samples were heated at 98 °C for 5 min, followed by SDS-PAGE for 45 min. The separated proteins were transferred electrophoretically to Immobilon-P PVDF membranes (Millipore, Billerica, MA, USA) for 45 min and membranes were blocked with 5% dried skimmed milk at 4 °C overnight. After blocking, membranes were incubated with primary antibodies against human L-type amino acid transporter 1 (hLAT1) polyclonal antibody rabbit (1:500; Trans Genic Inc., Kumamoto, Japan) or β-actin antibody rabbit (1:1000; Sigma-Aldrich) at room temperature for 1.5 h. After incubation, the membranes were washed TBST buffer (1 M Tris-HCl (pH 7.4), 5 N NaCl, Tween20 and H_2O), and incubated with ECL anti-rabbit IgG HRP-linked whole antibody donkey (1:10,000; GE Healthcare, Little Chalfont, UK) as the secondary antibody for 60 min. After washing with TBST buffer and TBS buffer (1 M Tris-HCl (pH 7.4), 5N NaCl and H_2O), the membranes were dipped in ECL Prime Western Blotting Detection Reagent (GE Healthcare). The protein expression was detected using an Image Quant LAS 4000 (GE Healthcare).

HeLa cells (2.5 × 10^4 cells per well) and HEK 293 cells (1 × 10^5 cells per well) were seeded in BioCoat™ Collagen I Cellware 4-Well Culture Slide in 1 mL of medium. After overnight incubation for HeLa cells and 2-day incubation for HEK 293 cells, the cells were rinsed once with PBS and fixed with 4% paraformaldehyde in PBS for 30 min. The fixed cells were rinsed with PBS and osmotically treated for 30 min at −20 °C in methanol, then incubated with human serum albumin (1:100; Sigma Aldrich) diluted in PBS for 30 min to block non-specific binding sites. Cells were rinsed three times with PBS and the primary antibody, anti-hLAT1 polyclonal antibody rabbit (1:100) was added in PBS at 4 °C overnight. Cells were rinsed three times with PBS and the secondary antibodies, Alexa Fluor® 488-labeled donkey anti-rabbit IgG antibodies (1:1000; Invitrogen) were added in PBS for 60 min at room temperature. After cells were rinsed three times with PBS, coverslips were mounted over cells in VECTORSHIELD® Hard·Set™ Mounting Medium with DAPI (Vector Labolatories, Burlingame, CA). Cells were then observed with a FV1000D (Olympus, Tokyo, Japan) confocal laser-scanning microscope.

As a result, the 38-kDa protein band, attributed to hLAT1, was detected in HeLa cells, while there was no band in HEK 293 cells. Anti-β-actin antibody was using as a loading control (Figure S3 (a)). The 125-kDa-protein band in HeLa cells is probably a dimer of LAT1 and 4F2hc [48]. Microscopy images of cells after treatment with an anti-hLAT1 polyclonal antibody also showed the expression of LAT1 on the membrane of HeLa cells, while expression was not observed in the fluorescent image of HEK 293 cells (Figure S3 (b)). These results demonstrate that HeLa cells express LAT1 while HEK 293 cells do not.

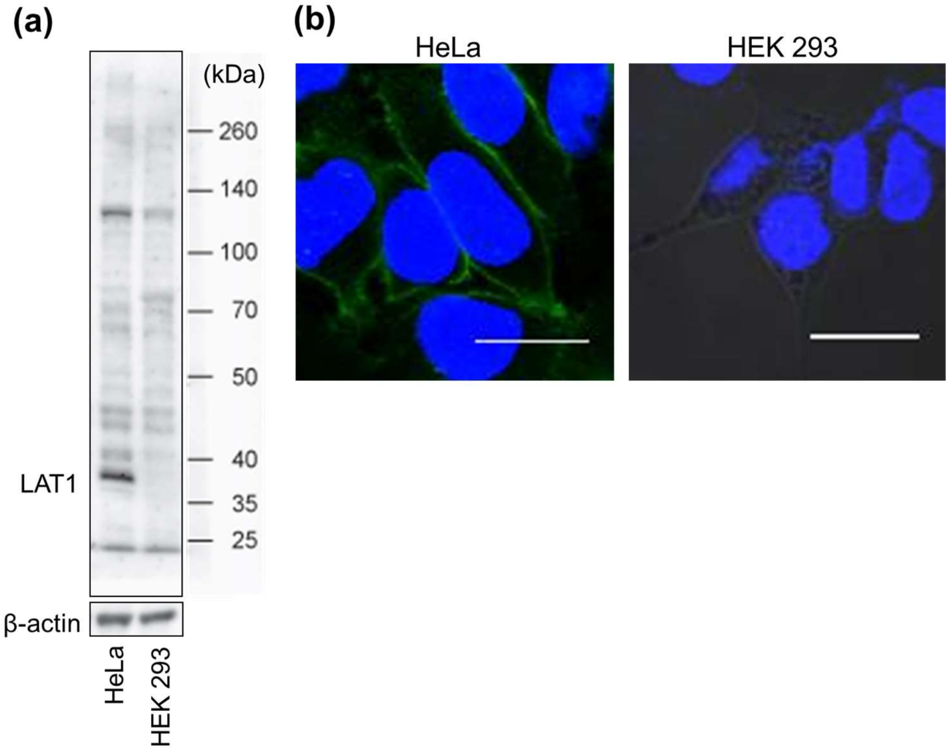

Figure S3. Western blot analysis was performed on the membrane fraction prepared from HeLa and HEK 293 cells using an anti-hLAT1 antibody (**a**). For HeLa cells, the 38-kDa-protein band was detected. An anti-β-actin antibody was used as a loading control. Microscopy images of HeLa and HEK 293 cells after treatment with an anti-hLAT1 polyclonal antibody (**b**). hLAT1 appears green and the nuclei were stained blue (DAPI). Magnification is 60×; scale bar represents 20 μm.

Fluorescence Intensity of Probes

Fluorescence spectra were measured using an FP-6300 spectrofluorometer (Jasco), and the temperature was controlled using an ETC-273T controller (Jasco) and a PT-31 Peltier system (Krüss, Hamburg, Germany). The maxima of the excitation wavelengths (λ_{ex}) and the emission wavelengths (λ_{em}) of the fluorescent probes were measured using concentrations of 1 mg/mL in PBS solution. The effect of temperature on the fluorescence intensity of the probes was evaluated between 25 °C and 45 °C.

The FL modification rate of the probes was determined from the absorption spectrum and the calibration curve of fluorescein-5-maleimide (FL) (Figure S4), and the maximum absorption wavelength values of P(NIPAAm-co-DMAAm20%)-FL and Tyr-P(NIPAAm-co-DMAAm20%)-FL (Figure S5). The excitation wavelengths and fluorescence wavelengths of the fluorescent probes were examined (Figure S6). λ_{ex} of P(NIPAAm-co-DMAAm20%)-FL was 492 nm, λ_{em} of (NIPAAm-co-DMAAm20%)-FL was 516 nm, λ_{ex} of Tyr-P(NIPAAm-co-DMAAm20%)-FL was 492 nm, λ_{em} of Tyr-P(NIPAAm-co-DMAAm20%)-FL was 517 nm. The terminal conjugation of fluorescein-5-maleimide to the polymers was possible while maintaining the characteristics of the fluorescent group. Changes in the fluorescence intensity of the fluorescent probes with temperature were observed. Since the polymers are hydrophilic and elongated below the LCST, the fluorescence intensity is presumed to be maintained. In contrast, the fluorescence intensity gradually decreased when the temperature rose by 2–3 °C above the LCST. It is considered that the fluorescent polymer becomes hydrophobic at the LCST or higher and further agglomeration proceeds by raising the temperature (Figure S7).

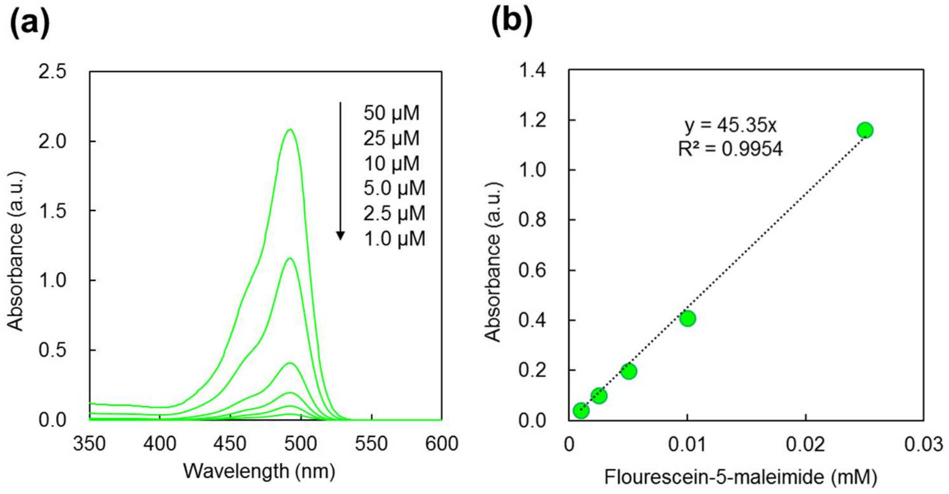

Figure S4. Absorption spectrum (**a**) and calibration curve (**b**) of fluorescein-5-maleimide.

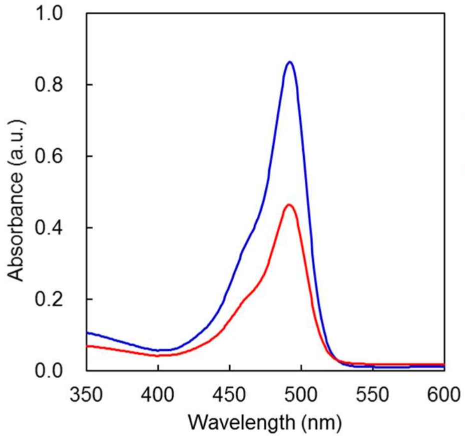

	Absorbance (a.u.)	Modification rate (%)
P(NIPAAm-*co*-DMAAm20%)-FL	0.864	57.2
Tyr-P(NIPAAm-*co*-DMAAm20%)-FL	0.465	30.8

Figure S5. Absorption spectra and modification rate of fluorescein-5-maleimide for P(NIPAAm-*co*-DMAAm20%)-FL and Tyr-P(NIPAAm-*co*-DMAAm20%)-FL.

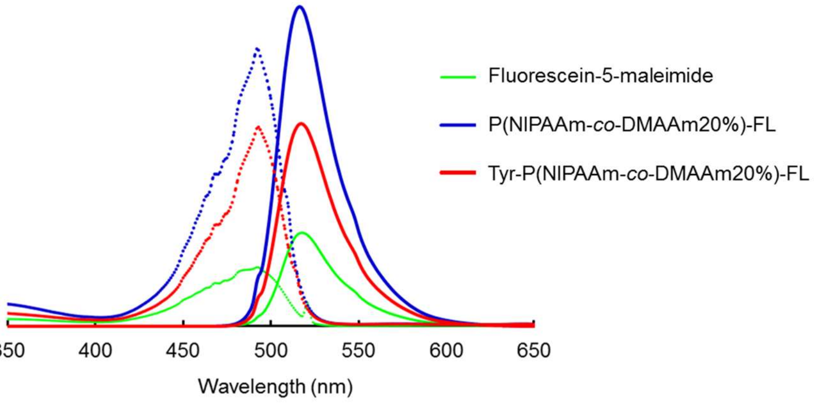

Figure S6. Excitation and emission spectra of fluorescent polymers and fluorescein-5-maleimide. Solid line: emission spectra, dotted line: excitation spectra.

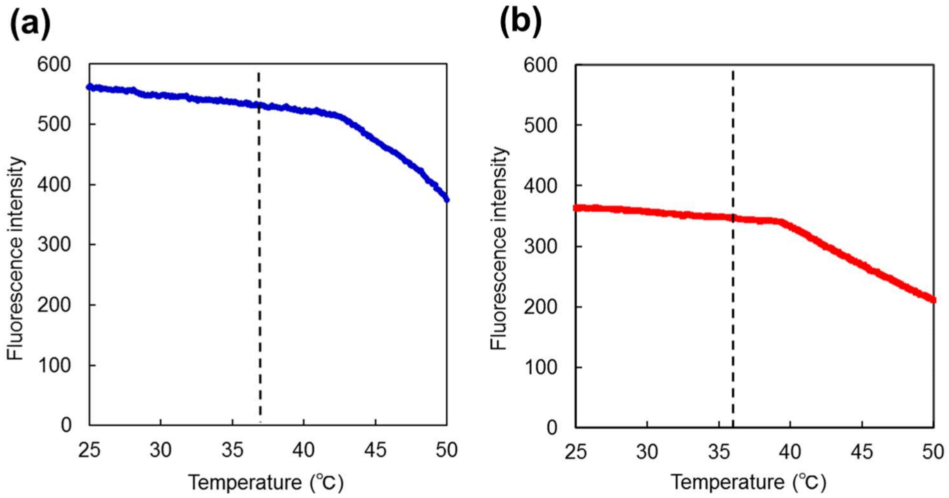

Figure S7. Temperature-responsive change of fluorescence intensity (**a**) P(NIPAAm-*co*-DMAAm20%)-FL and (**b**) Tyr-P(NIPAAm-*co*-DMAAm20%)-FL.

Reference

48. Yanagida, O.; Kanai, Y.; Chairoungdua, A.; Kim, D.K.; Segawa, H.; Nii, T.; Cha, S.H.; Matsuo, H.; Fukushima, J.; Fukasawa, Y.; et. al. Human L-type amino acid transporter 1 (LAT1): Characterization of function and expression in tumor cell lines. *Biochim. Biophys. Acta.* **2001**, *1514*, 291–302, doi:10.1016/S0005-2736(01)00384-4.
